# Fungal liver infection mimicking hepatic malignant tumor on contrast enhanced ultrasound: A case report

**DOI:** 10.1097/MD.0000000000025178

**Published:** 2021-04-02

**Authors:** Jie Yang, Ya-han Zhang, Jia-yan Huang, Qiang Lu

**Affiliations:** a Department of Ultrasound; b Department of Pathology, West China Hospital of Sichuan University, No. 37 Guo Xue Xiang, Chengdu, Sichuan Province, China.

**Keywords:** case report, contrast media, digestive system diseases, mycoses, ultrasonography

## Abstract

**Rational:**

Fungal liver infection mostly occurs in immunocompromised patients, and is often associated with delayed diagnosis and high mortality rates. Dynamic contrast enhanced imaging is crucial for the diagnosis of fungal liver infection and has been reported having variable manifestations.

**Patient concerns:**

A 38-year-old Chinese man, with a history of diabetes and chronic hepatitis B, was admitted to our hospital due to prolonged fever of unknown cause. He had a medical history of receiving broad-spectrum antibiotic treatment for pulmonary inflammation at the local hospital. The blood test results showed that the white cell count (14.0 × 10^9^/L) and neutrophil count ratio (77.0%) were subtly elevated. C-reactive protein (92.0 mg/l) and cancer antigen (CA)-125 (904.50 U/ml) were elevated. Non-small cell lung cancer antigen was within the normal limit. Hepatitis B virus DNA load was 3.28 × 10^3^ IU/ml. Sputum and blood cultures were normal. Abdominal ultrasonography (US) found a large heterogeneous mass, with diffused echogenic foci without infiltrating the surrounding vascular, which exhibiting “rapid wash in and out” on contrast-enhanced ultrasound (CEUS).

**Diagnosis:**

The diagnosis of liver fungal infection was confirmed pathologically via ultrasound-guided biopsy.

**Interventions:**

Antibiotic and antifungal therapy with imipenem and voriconazole.

**Outcomes:**

The patient's body temperature had been controlled and the huge mass disappeared on follow-up ultrasound 1-year later.

**Lessons:**

This case highlights the unusual imaging features of fungal liver infection, presenting as huge heterogeneous mass with diffusive echogenic foci without infiltrating the surrounding vascular on grayscale US and the enhancement pattern of “rapid wash in and out” on CEUS. Additionally, ultrasound-guided biopsy is necessary for the correct diagnosis of suspected liver lesions.

## 1 Introduction

A marked rise has been reported in the incidence of fungal hepatic infection in immunocompromised patients. Patients who are at risk of immunosuppression including those on immunosuppressive drugs after liver or other solid organ transplantation, those on anti-cancer chemotherapy, and those with uncontrolled diabetes mellitus or advanced cirrhosis.^[9,13]^ Ultrasonography (US) and computed tomography (CT) play an essential role in the diagnosis of hepatic fungal infection and in the follow-up of treatment.^[2,10,12]^ According to previous reports, the ultrasonographic pattern of liver fungal infection varies as the disease progresses, including the “wheel within wheel” phenomenon; “bull's eye” lesions; hypoechoic defects; and echogenic spots.^[1,2,5]^

To our knowledge, the reported cases mostly involved immunosuppressant patients^[3,4,8,12]^ Herein, we report a pathology-proven hepatic fungal infection case, mimicking hepatic malignant tumor, in a 38-year-old man with a history of diabetes and chronic hepatitis B. This case is helpful for the understanding of unusual imaging characteristics of hepatic fungal infection in patients at low risk of mycosis. Institution review board approved this case report and the requirement to obtain written informed consent was obtained.

## 2 Case report

A 38-year-old man had a 3-month history of prolonged fever of unknown cause, with occasional cough but without neutropenia, night sweats and abdominal pain. The patient was admitted to a local hospital 1 month prior, and chest CT suggested pulmonary inflammation. After broad-spectrum antibiotic treatment, the fever was not controlled well. Therefore, he was transferred to our hospital (a tertiary referring hospital) on December 1, 2015. The patient had a history of type II diabetes for 1 year and chronic hepatitis B for more than 10 years, without systematic antiviral treatment. Physical examination showed heart rate at 87 beats/minute, blood pressure 131/76 mm Hg, respiratory rate 20 breaths/minute, temperature 36.8°C, and found lung murmurs without enlargement of the liver and spleen. The blood test results showed that the white cell count (14.0 × 10^9^/L) and neutrophil count ratio (77.0%) were subtly elevated. C-reactive protein (92.0 mg/L) and cancer antigen (CA)-125 (904.50 U/ml) were elevated. Non-small cell lung cancer antigen was within the normal limit. Hepatitis B virus DNA load was 3.28 × 10^3^ IU/ml. Sputum and blood cultures were normal.

**Figure 1:**
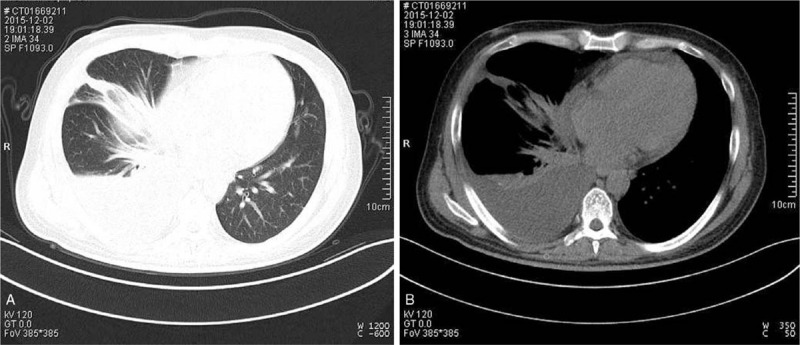
Imaging findings on computed tomography (CT). Chest CT found pleural effusion of the right thoracic cavity and thickening of the right pleura (A-B).

**Figure 2:**
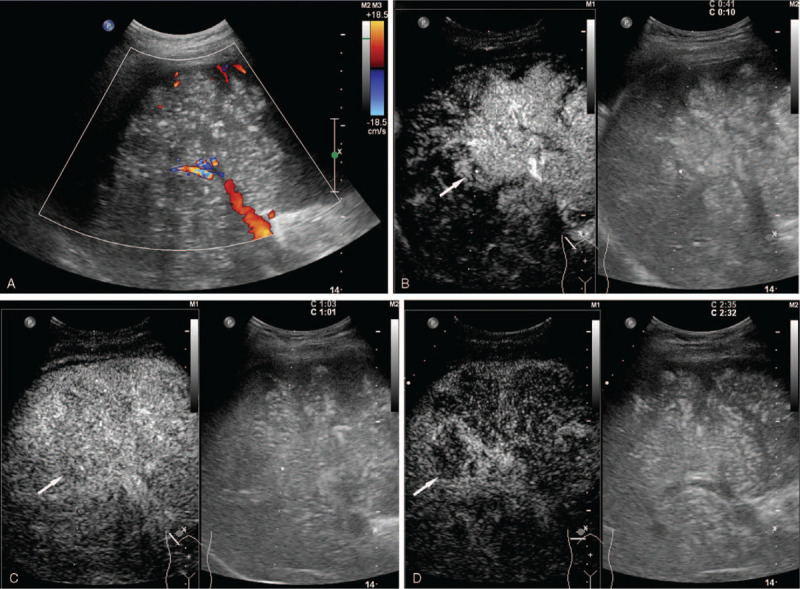
Imaging findings on contrast-enhanced ultrasound. (A) A solid heterogeneous mass with diffusive echogenic foci (“sesame biscuit” sign) measuring 17 cm is demonstrated by ultrasonography in a 38-year-old Chinese man with chronic hepatitis B. On color Doppler ultrasound, the mass encompassed the right portal vein without infiltrating the lumen. (B) Homogenous arterial hyperenhancement (white arrow) is illustrated on contrast enhanced (CE) US. (C-D) Early washout (white arrow) and mild washout (white arrow) were demonstrated in the portal and late phase, respectively. The lesion was classified as LR-M category according to the CEUS Liver Imaging Reporting and Data System, indicating malignancy but not specific to hepatocellular carcinoma. CEUS; contrast enhanced ultrasound.

Chest CT found pleural effusion of the right thoracic cavity and thickening of the right pleura (Fig. 1A-B). Pleural fluid was drained and increased lymphocytes were found in pathology analysis. Meanwhile, abdominal conventional US revealed a large heterogeneous mass with diffuse echogenic foci (“sesame biscuit” sign), measuring approximately 17 cm × 12 cm in size. On color Doppler imaging, the mass encompassed the portal vein without infiltrating the lumen (Fig. 2A). On contrast-enhanced ultrasound (CEUS) study, a bolus of 2 ml ultrasound agent SonoVue (Bracco) was injected through a 20-gauge line in the antecubital vein. The CEUS patterns indicated the diagnosis of hepatic malignancy and inflammatory disease (i.e., arterial phase hyperenhancement with early washout which is in line with the classification of LR-M according to the CEUS Liver Imaging Reporting and Data System) (Fig. 2B-D), and the history of hepatitis B infection increased the potential diagnosis of hepatocellular carcinoma.

**Figure 3:**
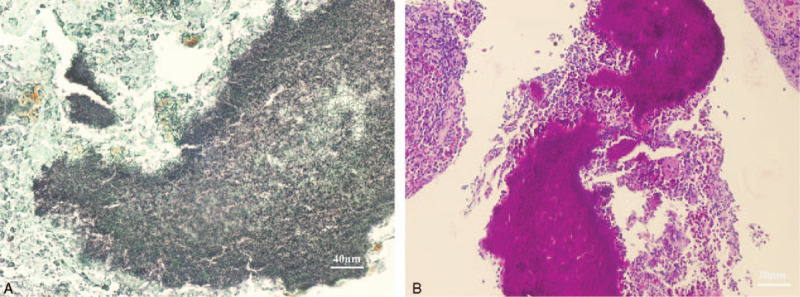
Histopathologic analysis of the biopsy specimen. After multidisciplinary discussion, the patient was referred to have US guided biopsy to obtain a definitive diagnosis. Fungal filaments are revealed by further staining with (A) Gomori methenamine silver (magnification × 400) and (B) periodic acid-Schiff stain (magnification × 200).

After multidisciplinary discussion, the patient was referred to have US guided biopsy to obtain a definitive diagnosis. The patient was referred for ultrasound-guided liver biopsy in the Department of Ultrasound. Microscopically, hematoxylin, and eosin staining revealed inflammatory changes with suspected coenobium. Special staining results: GMS (+), PAS (+), and the positive gram staining indicated mycelia (Fig. 3A-B). Hematoxylin and eosin staining revealed inflammatory changes with suspected coenobium.

**Figure 4:**
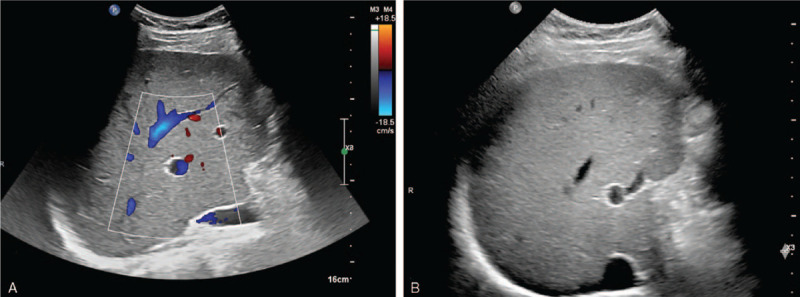
Follow-up liver ultrasound findings. One year later, follow up ultrasound confirmed the disappearance of the huge mass by illustrating homogeneous liver parenchyma in the right lobe where the huge hepatic fungal infection situated. (A-B).

Considering the patient's severe pulmonary infection and hepatic fungal infection, combination of antibiotic (imipenem, 500 mg, q8 h) and antimycotic drugs (voriconazole, 250 mg, q12 h) were used. After the treatment, the fever had been controlled well. The follow-up assessment showed that the patient had no fever recurrence. B-mode ultrasound demonstrated homogeneous liver parenchymal without mass 1-year later (Fig. 4A-B).

## 3 Discussion

A marked rise in the incidence of fungal hepatic infections has been reported, which occurs mainly in immunocompromised patients, and is often associated with delayed diagnosis and high mortality rates.^[9,13]^ However, there was insufficient clinical information and no typical clinical symptoms or serum biomarkers indicating the diagnosis of liver mycoses. Dynamic contrast enhanced imaging is crucial for the diagnosis of fungal liver infection and has been reported having variable manifestations. According to the literatures,^[1,2,5]^ ultrasonographic pattern of fungal liver infection varies as the disease progresses, including:

1. the “wheel within wheel” sign;
2. “bull's eye” sign;
3. hypoechoic defects; and
4. echogenic spots.

Pastakia et al^[1]^ discussed the imaging changes correlated with disease progression based on histological analysis. They reported that a “wheel within wheel” pattern was found in the acute phase of the disease. The peripheral hypoechoic rim was formed by a ring of fibrosis, while the echogenic zone (the inner wheel) was composed of tissue undergoing the inflammatory process. The center of the hypoechoic zone was associated with an area of necrosis with or without fungal elements. If there was no necrotic tissue, the patterns could present as “bull's eye”.^[2,6,7]^ They also reported that inflammation would disappear, as evidenced by the presence of hypoechoic defects if treated appropriately.^[2,6,7,11]^ In the late phase of the disease, the fibrosis components change into scar tissue, presenting as echogenic foci (areas of calcification as proven by pathology) with smaller sizes than the abovementioned lesion patterns.^[1,11]^ In this case, the scattered echogenic foci on conventional ultrasound may be related to the change of the multiple scar tissues within the mass.

To the best of our knowledge, the radiologic findings of fungal liver infection were rarely reported in patients without immunosuppression conditions. Yang et al^[2]^ reported 2 hepatic fungal infection cases in out-patients with a long history of taking antibiotics. These 2 cases showed similar US and CDFI features as those in at risk patients and appeared as multiple hypoechoic lesions smaller than 2 cm in diameter on US, and presented “filling defect” pattern in the whole CEUS process. However, in our case, abdominal conventional US revealed a large, inhomogeneous and poorly defined mass with diffusive echogenic foci, mimicking the appearance of a sesame biscuit, and the CEUS pattern manifested as liver malignancy (LR-M category). Besides the unusual imaging features, the history of chronic hepatitis B also increased the possibility of misdiagnosis of hepatocellular carcinoma. The un-infiltrated portal vein on conventional US and the history of antibiotic treatment may lead to the consideration of inflammatory lesions. The final diagnosis of liver fungal infection was made pathologically via ultrasound-guided biopsy.

## 4 Conclusion

Although fungal liver infection is rare in patient at risk for HCC, when a heterogeneous solid mass shows diffusive echogenic foci on conventional ultrasound and LR-M classification on CEUS, the diagnosis of fungal liver infection should be considered. Additionally, ultrasound-guided biopsy is necessary for the correct diagnosis of hepatic fungal infection.

## Author contributions

**Conceptualization:** Qiang Lu.

**Data curation:** Jie Yang, Ya-han Zhang, Jia-yan Huang.

**Funding acquisition:** Qiang Lu.

**Investigation:** Jie Yang.

**Methodology:** Qiang Lu.

**Resources:** Qiang Lu.

**Software:** Jie Yang.

**Supervision:** Qiang Lu.

**Visualization:** Ya-han Zhang.

**Writing – original draft:** Jie Yang.

**Writing – review & editing:** Jia-yan Huang, Qiang Lu.
